# Difference in trafficking of brain-derived neurotrophic factor between axons and dendrites of cortical neurons, revealed by live-cell imaging

**DOI:** 10.1186/1471-2202-6-42

**Published:** 2005-06-21

**Authors:** Naoki Adachi, Keigo Kohara, Tadaharu Tsumoto

**Affiliations:** 1 Division of Neurophysiology, Osaka University Graduate School of Medicine, 2-2 Yamadaoka, Suita, Osaka 565-0871, Japan; 2 Solution Oriented Research for Science and Technology, Japan Science and Technology Agency, Kawaguchi 442-0012, Japan

## Abstract

**Background:**

Brain-derived neurotrophic factor (BDNF), which is sorted into a regulated secretory pathway of neurons, is supposed to act retrogradely through dendrites on presynaptic neurons or anterogradely through axons on postsynaptic neurons. Depending on which is the case, the pattern and direction of trafficking of BDNF in dendrites and axons are expected to be different. To address this issue, we analyzed movements of green fluorescent protein (GFP)-tagged BDNF in axons and dendrites of living cortical neurons by time-lapse imaging. In part of the experiments, the expression of BDNF tagged with cyan fluorescent protein (CFP) was compared with that of nerve growth factor (NGF) tagged with yellow fluorescent protein (YFP), to see whether fluorescent protein-tagged BDNF is expressed in a manner specific to this neurotrophin.

**Results:**

We found that BDNF tagged with GFP or CFP was expressed in a punctated manner in dendrites and axons in about two-thirds of neurons into which plasmid cDNAs had been injected, while NGF tagged with GFP or YFP was diffusely expressed even in dendrites in about 70% of the plasmid-injected neurons. In neurons in which BDNF-GFP was expressed as vesicular puncta in axons, 59 and 23% of the puncta were moving rapidly in the anterograde and retrograde directions, respectively. On the other hand, 64% of BDNF-GFP puncta in dendrites did not move at all or fluttered back and forth within a short distance. The rest of the puncta in dendrites were moving relatively smoothly in either direction, but their mean velocity of transport, 0.47 ± 0.23 (SD) μm/s, was slower than that of the moving puncta in axons (0.73 ± 0.26 μm/s).

**Conclusion:**

The present results show that the pattern and velocity of the trafficking of fluorescence protein-tagged BDNF are different between axons and dendrites, and suggest that the anterograde transport in axons may be the dominant stream of BDNF to release sites.

## Background

Neurotrophins have been considered to play roles in the differentiation, neurite outgrowth and survival of a certain group of neurons [1-4]. In addition to these well-known functions, most neurotrophins are involved in rapid changes in the function of neural circuits [5,6]. In particular, brain-derived neurotrophic factor (BDNF) plays a role in activity-dependent changes in synaptic function [7-9].

To serve such a broad-ranging function, BDNF produced in the nucleus of neurons is sorted into a regulated secretory pathway through the *trans*-Golgi network, and transported to release sites in neurites [10,11]. In axons it is suggested that BDNF is transported through the fast axonal flow and then released and transferred to postsynaptic neurons in an activity-dependent manner [10-15]. In dendrites or dendrite-like neurites also, the targeting of BDNF to distal parts and its release were suggested to occur in an activity-dependent manner [16-22]. Since these previous studies were carried out using immunohistochemical and/or *in situ *hybridization technique after the fixation of neurons, or by observing the decrease in fluorescence intensity of neurons expressing BDNF tagged with green fluorescent protein (GFP), the actual dynamics of BDNF trafficking was not analyzed in dendrites. Thus, a question of whether the trafficking of BDNF is different between axons and dendrites of neurons is not answered yet.

An answer to this question will give a clue that may resolve the controversial issue of whether BDNF acts retrogradely through dendrites on presynaptic neurons or anterogradely through axons on postsynaptic neurons. To address this question, it is desirable to perform a real-time analysis of movements of BDNF tagged with GFP in both axons and dendrites of living neurons. However, such an analysis of BDNF trafficking has not been successfully carried out except for two recent studies on its retrograde transport in dorsal root ganglion neurons [23] and bidirectional transport in cortical cell neurites [24]. However, the observation was restricted to axons in the former study, and axons and dendrites were not distinguished in the latter study.

In the present study we carried out a real-time analysis of movements of BDNF tagged with GFP in both axons and dendrites of living cortical neurons using the method of direct injection of their plasmid cDNAs into the nucleus [14]. We found that most of BDNF-GFP moves smoothly in the anterograde direction in axons while it does not move in dendrites in most cases. Even if it moves in dendrites, its velocity is slower than that in axons.

Parts of the present results were published in an abstract form [25].

## Results

Plasmids encoding BDNF tagged with GFP or CFP at the COOH-terminus were injected into the nucleus of cultured cortical neurons through a micropipette under visual control, as reported previously [14]. GFP- or CFP-tagged BDNF resulting from these plasmids were confirmed to be biologically active and mimic the releasing properties of untagged BDNF [20]. In the present experiments, 20–30% of the neurons into which plasmids had been injected expressed fluorescent signals, as reported previously [14]. The expression of signals was already detected 16 h after the injection, but recordings were usually carried out about 24 h after the injection.

**Figure 1:**
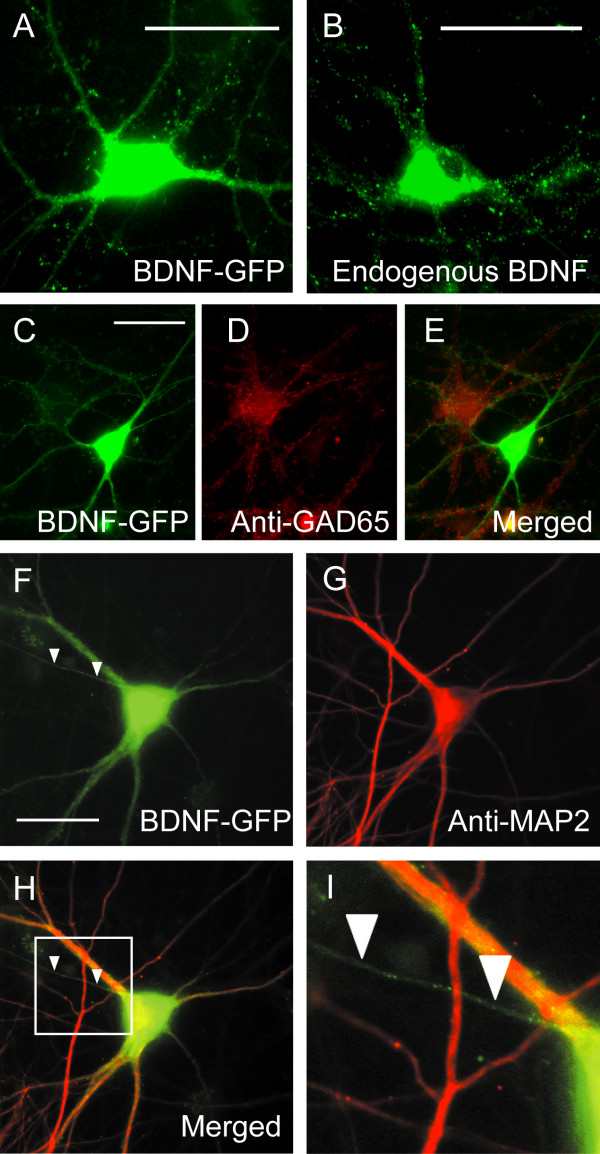
Similar distribution of BDNF-GFP and endogenous BDNF, and expression of BDNF-GFP in GAD65-negative neurons and its localization in axons and somatodendritic regions. **A**, BDNF-GFP expressed in a neuron 24 h after plasmid cDNA encoding BDNF-GFP was injected into the nucleus. Scale bar in A and B indicates 50 μm **B**, Immunocytochemical image of another, non-injected neuron stained with anti-BDNF antibody. **C**, Image of neurons expressing and not-expressing BDNF-GFP. Scale bar indicates 50 μm and applies to D and E. **D**, Image of the neurons shown in C, immunocytochemically stained with anti-GAD65 antibody. **E**, Superimposed images of C and D. **F**, Distribution of BDNF-GFP expressed in another neuron. Arrowheads indicate a MAP2-negative neurite. Scale bar indicates 50 μm and applies to G and H. **G**, Image of the same neuron as shown in F, immunocytochemically stained with anti-MAP2 antibody. **H**, Superimposed images of F and G. **I**, Magnified image of the boxed area in H. Vesicular puncta of BDNF-GFP in the axon are seen more clearly than those in H.

In about two-thirds of the neurons that expressed fluorescent signals of BDNF-GFP, the signals were punctated in clusters along dendrites and axons (Fig. 1A). The soma and bases of dendritic trunks appeared to be filled with fluorescent signals because of the very dense expression of BDNF-GFP. To see whether the distribution of BDNF-GFP signal was similar to that of endogenous BDNF, neurons cultured in companion dishes (sister-cultured neurons) were stained immunocytochemically with antibody to BDNF. The distribution of endogenous BDNF was also punctated in neurites and diffuse in the soma (Fig. 1B), as reported previously (14, 19, 20). This distribution pattern was similar to that of granular BDNF-GFP, suggesting that BDNF-GFP was expressed in plasmid-injected neurons in a similar way to endogenous BDNF if the expression in neurites was granular. In about one-third of the neurons that expressed fluorescence, the signals were diffuse and smear-like even in dendrites. These neurons were not included in the analysis of BDNF-GFP trafficking.

In the present study we attempted to inject plasmids into pyramidal cell-like neurons which are excitatory. To confirm that neurons expressing fluorescent signals were excitatory, they were stained immunocytochemically with anti-glutamic acid decarboxylase 65 (GAD65) antibody. As shown in Figure 1C and 1D, a neuron that expressed BDNF-GFP was not stained with the antibody. This was confirmed by the superposition of the two images (Fig. 1E). This result suggests that the BDNF-GFP-injected cell was an excitatory neuron. All of the 12 BDNF-GFP-injected neurons that were immunocytochemically tested were negative to anti-GAD65 antibody, indicating that the neurons analyzed in the present study were most likely excitatory. We also stained neurons by immunocytochemistry using anti-microtubule-associated protein 2 (MAP2) antibody to differentiate axons from dendrites. As shown in Figure 1F–I, we could identify axons on the basis of their negativity to anti-MAP2 antibody (arrowheads in Fig. 1F, H and 1I). Fluorescent signals were detected also in MAP2-negative neurites (Fig. 1H and 1I), indicating that BDNF-GFP was expressed in axons as well as somatodendritic regions.

**Figure 2:**
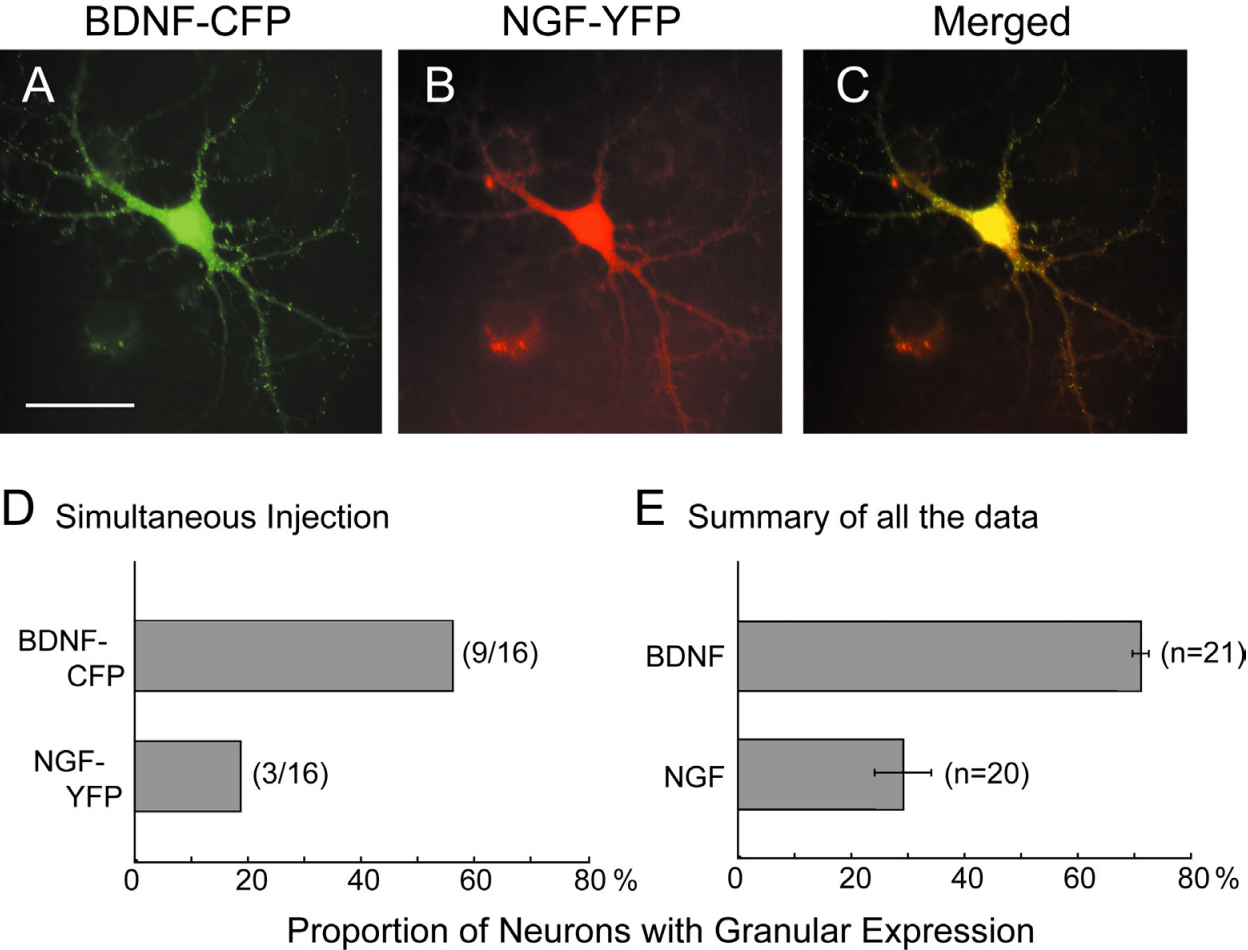
Simultaneous expression of BDNF-CFP and NGF-YFP in the same neuron and proportion of neurons with granular expression of fluorescent puncta. **A**, Image of a neuron expressing BDNF-CFP. Scale bar indicates 50 μm and applies to B and C. **B**, Image of the same neuron as in A, expressing NGF-YFP. **C**, Superimposed images of A and B. **D**, Proportion of neurons with granular expression of fluorescent puncta. Numerators at the right end of bar indicate the number of neurons showing granular expression of each neurotrophin tagged with CFP or YFP, and denominators indicate the total number of cells which expressed both fluorescent signals. **E**, Mean proportions of neurons with granular expression of fluorescent puncta, obtained from all the data including those obtained with the single injection of plasmid cDNAs. Numbers at the right end of bar indicate the number of injection trials, in which 4–10 neurons expressed fluorescent signals. Horizontal lines at the end of bars indicate 2SEMs.

In part of the experiments we injected plasmids encoding NGF-GFP to see whether BDNF-GFP is expressed in a manner specific to this neurotrophin. The granular expression of NGF-GFP was seen in a minor group of the cells that expressed fluorescent signal (see Fig. 2E). In most of the cells NGF-GFP was expressed in a smear-like manner even in dendrites. To exclude a possibility that such a difference in the expression pattern between the two neurotrophins tagged with GFP might reflect cell-to-cell variability, we simultaneously injected a combination of plasmids, BDNF-CFP and NGF-YFP into the nucleus of a single neuron. For this purpose CFP or YFP was used in place of GFP, because the fluorescent wavelength of GFP overlaps with that of CFP or YFP, but those of CFP and YFP do not substantially overlap with each other. As in the case of single injection, BDNF-CFP was expressed mostly in a granular manner while NGF-YFP was expressed in a smear-like manner in the same cell. An example for this finding is shown in Figure 2A–C. Many puncta of BDNF-CFP were seen along neurites while NGF-YFP was seen in a smear-like manner in the neurites of the same cell. Such a difference in expression pattern between the two neurotrophins was evident in the superimposed figure (Fig. 2C). The results obtained with simultaneous expression of the two neurotrophins are summarized in Figure 2D. As seen in this graph, about 60% of the cells expressed CFP-tagged BDNF in a vesicular manner while only about 20% of the cells expressed YFP-tagged NGF in such a manner.

As mentioned before, the single injection of plasmid cDNAs encoding GFP-, CFP- or YFP-tagged neurotrophins also showed the difference in the expression pattern between BDNF and NGF (Fig. 2E). In this analysis the proportion of cells with vesicular expression was calculated for each session of injection trials and then the mean values were calculated from 21 and 20 sessions of trials for fluorescent protein-tagged BDNF and NGF, respectively. The means ± SEMs were 71.2 ± 1.5 and 29.2 ± 5.0%, respectively. The difference between these two values was statistically significant (P <0.001, unpaired t-test). These results indicate that the granular expression of BDNF-GFP was not common to other neurotrophins, although we did not observe the expression of fluorescent protein-tagged neurotrophin-3 and neurotrophin-4/5 in the present study.

**Figure 3:**
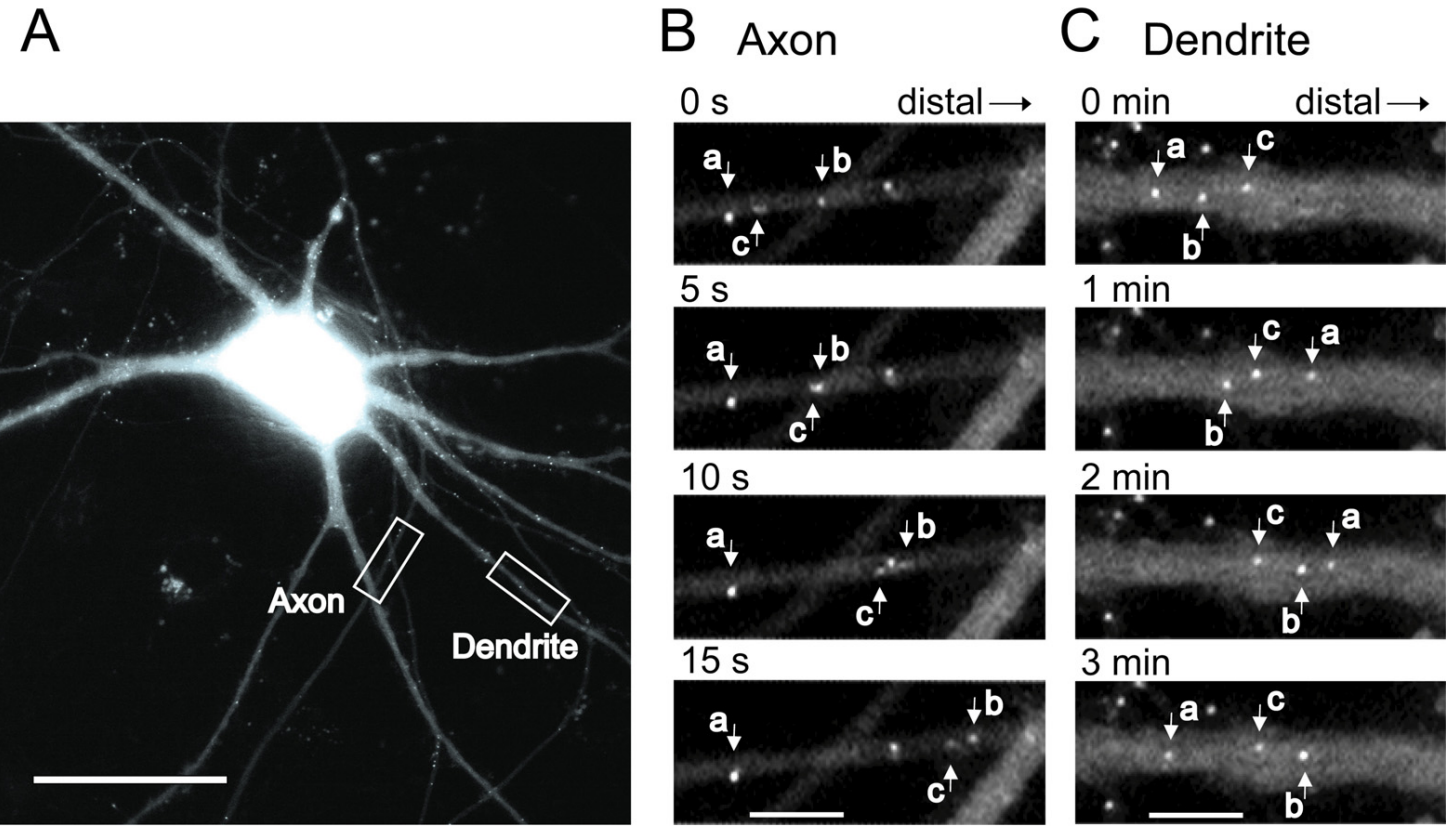
Movements of BDNF-GFP puncta in an axon and dendrites of a living neuron. **A**, Image of a cortical neuron which expressed BDNF-GFP in soma, axon and dendrites. Time-lapse images in each square along an axon and a dendrite are magnified and shown in B and C, respectively. Scale bar, 50 μm. **B**, Moving (b and c) and non-moving (a) puncta in the axon. Each image was taken at a time point indicated at the top of each figure. Right is the distal side of the axon. Scale bar in the bottom image indicates 5 μm and applies to all the images in B and C. **C**, Moving (a and b) and non-moving (c) puncta in the dendrite. Right is the distal side of the dendrite. Each image was taken at a time point indicated at the top of each figure.

We then found that most of the fluorescent puncta were moving in neurites of living neurons in the anterograde as well as retrograde directions (see Additional file 1). The movements of puncta were observed in about 80% of the neurons that showed a vesicular expression of BDNF-GFP. Neurons in which moving puncta were not detected were excluded from the analysis of BDNF-GFP trafficking. A detailed observation indicated that most of the puncta in axons moved smoothly in the anterograde direction, while most of the puncta in dendrites did not move or moved back and forth within a small distance in a fluttering manner. Figures 3B and 3C show an example of such observations. In an axon of a neuron shown in Figure 3B, a bright vesicle was observed to move in the anterograde direction at an almost constant velocity (punctum c) and another vesicle (punctum b) started to move about 10 s later (see also time-lapse pictures in Additional file 1). A small number of vesicles did not move during the observation time (punctum a). In dendrites, a substantial number of vesicles did not move (punctum c of Fig. 3C), but some puncta suddenly moved in the distal direction and then moved back to the original point (punctum a). We also found that a few puncta in dendrites moved initially, and then suddenly stopped and stayed in the same position during the observation time, as shown in Figure 3C (punctum b). These observations were carried out up to about 200 μm from the soma. Within this distance we did not detect a notable regional difference in the trafficking of puncta in dendrites nor in axons.

**Figure 4:**
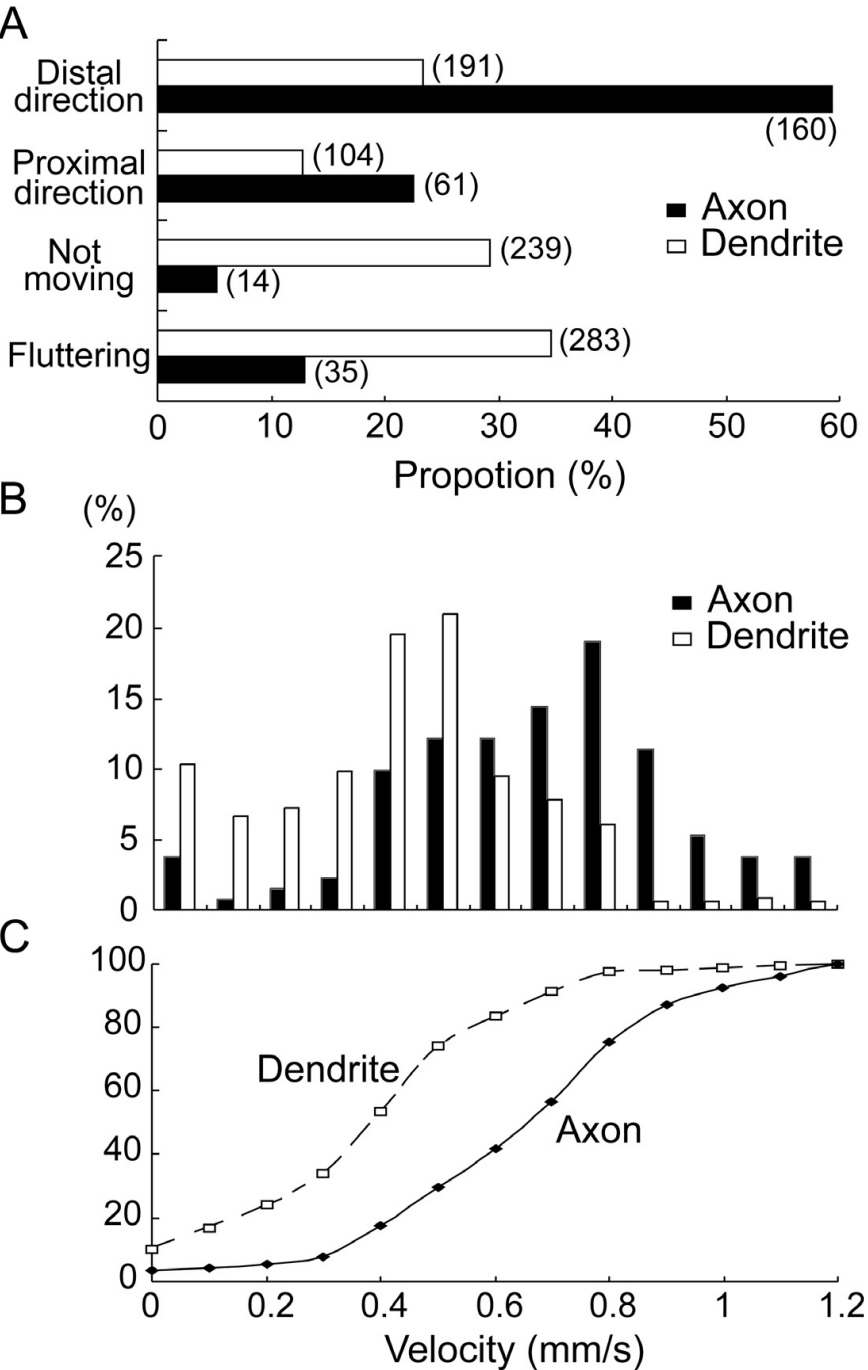
Properties of movement of BDNF-GFP puncta in axons and dendrites. **A**, A proportion of puncta which move in axons (filled columns) or dendrites (open columns) in the distal and proximal directions, do not move, and flutter within a small distance (10 μm). The numbers of puncta observed are given in parentheses. **B**, Distribution of moving puncta with different velocities. The bin width of the abscissa was 0.1 μm/s, and 0 indicates that the velocity was slower than 0.1 μm/s. The total numbers of vesicles analyzed were 132 in axons and 349 in dendrites. **C**, Cumulative plots of velocity distributions shown in the histograms of B. Dotted and continuous lines represent dendrites and axons, respectively.

To quantify these observations we classified fluorescence-positive puncta into four groups, i.e., puncta moving in the distal direction over a distance of 10 μm, those moving in the proximal direction over 10 μm, those not moving during the observation time of at least 15 min, and those fluttering within 10 μm. As shown in Figure 4A, 59% of the puncta in axons moved in the distal direction toward the terminals and only 23% of the puncta in axons moved in the proximal direction. On the other hand, 64% of the puncta in dendrites did not move at all or only fluttered within the short distance. Nevertheless, the rest of them in dendrites were observed to move in the distal or proximal direction. We then calculated the velocity of moving puncta in axons and dendrites, and found that the velocity was faster in axons on average than in dendrites. Figure 4B shows the distribution histograms of the velocity of moving puncta in axons or dendrites. The mean velocity of moving puncta in the distal (anterograde) direction was not markedly different from that in the proximal (retrograde) direction. Therefore, movements in both directions were combined in these histograms. The mean velocity in axons was 0.73 ± 0.26 (SD) μm/s, while that in dendrites was 0.47 ± 0.23 μm/s. The difference between these two distributions is evident in the cumulative probability curves of the values (Fig. 4C). The difference between the two curves was statistically significant (Kolmogorov-Smirnov test, P < 0.001).

## Discussion

In the live-cell imaging of GFP- or CFP-tagged BDNF, we observed that most of fluorescent protein-tagged BDNF which were expressed as vesicular puncta in axons moved rapidly in the anterograde direction, as reported previously [14]. It is possible to argue that because of the direct injection of the plasmids into the nucleus of neurons, the anterograde movements of BDNF-GFP from the soma were dominant. This possibility seems unlikely, however, because the movements of BDNF-GFP vesicles in axons were already observed 16 h after the injection and the vesicles were calculated to move over a distance of 20 mm by the observation time (24 h after the injection) assuming that the mean velocity of 0.73 μm/s was maintained. Thus, BDNF-GFP is expected to have reached terminals of neurites long before the observation time. Indeed we observed that a substantial proportion of vesicles were moving in the retrograde direction in axons and dendrites, as shown in Figure 4A.

BDNF-GFP vesicles were expressed in dendrites as well as in axons. The number of expressed vesicles in dendrites was much larger than that in axons. This difference seems to reflect the difference in areas between dendrites and axons. Thus, we did not find a strongly polarized expression of BDNF-GFP in either structural domain of neurons. In dendrites, however, the trafficking pattern of BDNF-GFP was markedly different from that in axons. Most of the BDNF-GFP vesicles in dendrites did not move at all or just fluttered within a small distance. Only a small number of puncta in dendrites moved continuously, while most of the puncta in axons moved smoothly over a long distance. There is a possibility that the directed movement of the fluorescent vesicles was not seen in dendrites, because BDNF-GFP had already arrived at dendritic sites where it was packed for release machinery. This possibility seems unlikely, since we did not observe a higher mobility of the puncta in dendrites at 16 h after the injection than at the standard observation time (24 h after the injection).

We further found that the velocity of transport of a minor group of BDNF-GFP puncta in dendrites was slower than that of BDNF-GFP puncta in axons. The mean velocity in axons (0.73 ± 0.26 μm/s) is comparable to the reported values for the anterograde transport of a synaptic vesicle protein, synaptophysin tagged with GFP, in the axons of mouse dorsal root ganglion cells (0.69 ± 0.33 μm/s, [26]) and of ^125^I-labelled BDNF in the optic nerve of neonatal rats (0.47–0.92 μm/s, [15]). The present observations that the velocity and the pattern of movements of BDNF-GFP in dendrites were different from those in axons seem consistent with the previous results that dendrites have microtubules of mixed polarity orientation, while axonal microtubules have a uniform polarity orientation, with their plus ends toward the axonal terminal [27] and that motor proteins, microtubule-based kinesin superfamily proteins (KIFs), are differentiated in axons and dendrites [28]. The question of what kinds of KIFs or other motor proteins are responsible for the difference in the pattern of trafficking of BDNF-GFP between axons and dendrites will be clarified in future studies.

In the present study using the plasmid cDNA injection method, there is a possibility that BDNF-GFP was expressed at much higher level than that of endogenous BDNF, and thus the sorting and trafficking characteristics would be different from those of endogenous BDNF. Although we cannot completely exclude this possibility, we believe that it is unlikely, based on the two observations. 1) The intracellular localization of expressed BDNF-GFP is similar to that of endogenous BDNF when BDNF-GFP is expressed in a punctated manner. 2) As discussed above, the mean velocity of trafficking of BDNF-GFP in axons is similar to the reported value for the anterograde transport of ^125^I-labelled BDNF in the optic nerve of neonatal rats. This suggests that the trafficking properties of BDNF-GFP are not significantly different from those of endogenous BDNF. Furthermore we observed that the expression pattern of BDNF-GFP or -CFP was different from that of NGF-GFP or -YFP in most cases. These results altogether suggest that the expression and trafficking of BDNF-GFP may not be substantially different from those of endogenous BDNF. This further suggests that the GFP tag may not notably affect sorting and trafficking of secretory proteins, as reported previously [26].

In sum, the present finding that the anterograde trafficking of BDNF-GFP in axons was dominant in cortical neurons seems consistent with the view that BDNF acts on postsynaptic neurons in the anterograde direction [13-15,29-35].

## Conclusion

The present results show that the pattern and velocity of the trafficking of BDNF are different between the two structural domains of neurons, axons and dendrites, and suggest that the anterograde transport in axons may be the dominant stream of BDNF to release sites.

## Methods

### Culture preparation of neurons

Neonatal mice (C57/BL6, postnatal day 0–1) were anesthetized with ketamine (> 30 mg/kg, i.p.), and then killed by cervical dislocation. The experimental procedures met the regulation of the Animal Care Committee of Osaka University Graduate School of Medicine. Neurons were cultured at a low density using conventional methods, as reported previously [14]. A piece of the visual cortex was removed from neonatal mice, enzymatically dissociated with papain (20 U/ml), and triturated with a fire-polished glass pipette. Neurons were plated on a previously prepared glial feeder layer, and were grown in a solution based on the Neurobasal A medium (GIBCO, Rockville, MD, USA) supplemented with 5% B27 (GIBCO). All experiments were carried out 14–24 days after plating.

### Injection of plasmid cDNA of BDNF or NGF tagged with fluorescent proteins

The cDNAs of mouse BDNF or NGF tagged with GFP, CFP or YFP at the C-terminus were provided by Dr. Masami Kojima. Glass micropipettes were filled with Tris-ethylene-diamine-tetraacetic acid (EDTA) buffer (pH 8.0) which contained cDNAs of BDNF-GFP, or -CFP (0.5–1 μg/μl), or NGF-GFP, or -YFP (0.5 μg/μl). Then cultured neurons were placed on the stage of an inverted epifluorescence microscope (TE300, Nikon, Tokyo, Japan), and cDNAs were injected into the nucleus of a neuron through a micropipette under visual control, using a micromanipulator (MMO-202ND, Narishige, Tokyo, Japan), as described previously [14].

### Live-cell imaging

Neurons which expressed fluorescent protein-tagged BDNF or NGF signals were observed using a 40 X /1.3 NA oil immersion objective (Plan Flour, Nikon) attached to the inverted epifluorescence microscope. The fluorescence of GFP. CFP and YFP excited by light at the wavelength of 488, 434 and 514 nm was measured using a cooled CCD camera (ORCA-ER, Hamamatsu Photonics, Hamamatsu, Japan). Time-lapse recordings were carried out about 24 h after the transfection, and sequential images were acquired using a cooled CCD camera at about 30°C. The exposure time was 1 or 2 s, the lapse of time was about 5 s, and the total time of recordings was 15–20 min. The trafficking of BDNF was analyzed by tracking the positions of GFP fluorescence-positive vesicles in neurites as a function of time. The analyses and animations of the data were made using the AQUA COSMOS software (Hamamatsu Photonics).

### Immunocytochemistry

For immunocytochemical staining, neurons were fixed with 4% paraformaldehyde (Sigma, St. Louis, MO, USA) and 4% sucrose in Dulbecco's phosphate-buffered saline (PBS) for 20 min at room temperature. The cells were incubated with PBS containing 0.2% Triton-X (Sigma) for 5 min and blocked by 10% goat serum in PBS for 1 h at 37°. Then, anti-MAP2 monoclonal antibody (isotype: IgG1, 1:250, Sigma), anti-BDNF polyclonal antibody (2 μg/μl, provided by Dr. Ritsuko Katoh-Semba), and anti-GAD65 monoclonal antibody (isotype IgG2a, 1:100, Chemicon, Temecula, CA, USA) were applied overnight at 4°. Endogenous BDNF was visualized by anti-rabbit secondary antibody conjugated with Alexa 546 (1:2000, Molecular Probe). MAP2 and GAD65 were visualized by anti-mouse secondary antibody conjugated with Alexa 647 or 350 (1:200, Chemicon), and by isotype-specific secondary antibody conjugated with Alexa 546 (1: 2000, Chemicon).

## Abbreviations

BDNF, brain-derived neurotrophic factor

CFP, cyan fluorescent protein

GAD65, glutamic acid decaroxylase 65

GFP, green fluorescent protein

KIF, kinesin superfamily protein

MAP2, microtubule-associated protein 2

NGF, nerve growth factor

PBS, phosphate-buffered saline

YFP, yellow fluorescent protein

## Authors' contributions

NA designed and carried out most of the experiments. KH helped with the design and analysis of experiments. TT planned the experiments and coordinated the work.

## Supplementary Material

Additional File 1 Supplementary movie of BDNF-GFP puncta in an axon and dendrites of a cortical neuron. An arrow at rest indicates an example of non-moving and fluttering puncta in dendrites, and another moving arrow that appeared 6 min after starting the movie indicates an example of fast-moving puncta in axons. Click here for file
